# Supplementary material for: The resilient potential behaviours in an Internal Medicine Department: Application of resilience assessment grid
Source: PLoS One. 2022 Oct 17;17(10):e0276178. doi: 10.1371/journal.pone.0276178 (PMC9576065; doi:10.1371/journal.pone.0276178)
Supplement: S1 Appendix — (DOCX) [file pone.0276178.s001.docx]

| **Themes** | **Questions** | **Notes** |
| --- | --- | --- |
| **Briefing** | [Introduction] Thank you for taking the time for this interview. The interview is part of a ph.d. project. We are gathering information on how the work is organised at the internal medicine department. The information will be used to construct a resilience of the department.  I am a Ph.D. student at the University Hospital of Southern Denmark, and welcome your views, feelings, and experiences. We are very interested in your honest opinions, and there are no right or wrong responses. This interview should take between 40 minutes to 1-hour but as there are a range of topics that I would like to cover, I may need to move the interview along at different points along the way. Your comfort is of utmost importance. As a reminder, you are free to pause and stop this interview at any time, without consequence. Do you have any questions so far? Can I confirm you have read the Pre-interview Information Sheet? [if not, the interviewer will go through this sheet now].  All the information that you give us will be kept confidential and any reports of the information will be completely anonymous. | |
| **Background** | 1. Can you please introduce yourself and tell a little about your work and responsibilities? 2. How long have you worked as a physician/nurse/manager in the department?(work experience) 3. Can you tell a little about what characterizes your daily work in the department? What types of tasks do you handle? | |
|  | | |
| **Respond** | - How do you respond if something unexpected happens? For example, an interruption, a pause required by a more urgent task that takes priority, a missing resource, etc. - Do you remember back to the last time you felt pressured to work? Can you describe how it was handled - Who can you talk to if you feel pressured? Does it help? - How is the collaboration with your colleagues? (internally and cross specialty) - Do you have delays in your work and how is it handled? |  |
| **Monitor** | - How do you gain overview of your daily work? What kind of indicators do you look at? - How do you know that the pressure is increasing? - How do you know when your colleagues need your help - Do you know what your colleagues do, what they need from you, and what their competencies can be used for? |  |
| **Learn** | - How does learning happen at the department? - Is learning ongoing or is it event driven? Is learning a part of your everyday work? - How do you share your experiences with your colleagues at the department? - Do you learn from things that go well in addition to things that did not go well? - Do you feel safe asking about something you don’t know? |  |
| **Anticipate** | - How do you get prepared for difficult or unforeseen situations? - Does the department actively work on identifying future opportunities? - Is the department aware of where they have challenges? - Does the department have the competences to carry out the work? - How are plans communicated at the department? |  |
| **leadership** | What do you need from your manager in relation to performing your everyday work? |  |
| **De-briefing** | Has anyone got any questions?  If you have any thoughts or questions please email me.  Thank you very much for your time |  |
