# Supplementary material for: The resilient potential behaviours in an Internal Medicine Department: Application of resilience assessment grid
Source: PLoS One. 2022 Oct 17;17(10):e0276178. doi: 10.1371/journal.pone.0276178 (PMC9576065; doi:10.1371/journal.pone.0276178)
Supplement: S2 Appendix — (PDF) [file pone.0276178.s002.pdf]

Det ambulante forløb i Medcinske Sygdomme Sønderborg/Tønder

Venligst udfyld nedenstående spørgeskema

Det ambulante forløb i Medicinske Sygdomme Sønderborg.

Tak fordi du vil deltage i spørgeskemaundersøgelsen. Det vil tage ca.10 min.

Undersøgelsen er en del af mit ph.d.-projekt, der har til formål at undersøge, hvordan arbejdet i det ambulante forløb er organiseret fra sundhedspersonalets perspektiv. Ph.d.-projektet er forankret i Medicinske Sygdomme Sønderborg/Tønder og bliver udført af ph.d. studerende Mariam Safi. Frans Brandt Kristensen er hovedvejleder for projektet.

Formålet med spørgeskemaundersøgelsen er, at få dybere indsigt i, hvordan arbejdets udførelse i det ambulante forløb i Medicinske Sygdomme Sønderborg bliver tilpasset til forholdene. Derfor er jeres perspektiv om arbejdet i det ambulante forløb meget vigtigt.

Resultaterne vil blive præsenteret til jer, når vi har modtaget alle besvarelserne.

Undersøgelsen er anonym og jeres personlige oplysninger kommer ikke til at fremgå nogle steder.

Har i nogle spørgsmål kan i kontakte mig på : 28 51 25 07 eller Mariam.Safi2@rsyd.dk

Baggrunds information

1) Hvad er din funktion?

◯ Afdelingssygeplejerske eller specialeansvarlig overlæge
◯ Sygeplejerske
◯ Læge

2) Hvor mange år har du arbejdet i Medicinske Sygdomme ambulatorie SØ/TØ?

◯ 0-1
◯ 1-3
◯ 3-5
◯ 5-10
◯ 10+

Respondering

		Aldrig	Sjældent	Somme tider	Ofte	Altid
3)	Der er tidsmæssigt fleksibilitet i mit ambulatorie program.	◯	◯	◯	◯	◯

4)

	I afdelingen hjælper vi hinanden i pressede situationer.	○	○	○	○	○
5)	I afdelingen kan vi varetage hinandens funktioner indenfor samme faggruppe.	○	○	○	○	○
6)	I afdelingen har vi en fælles forståelse for hvad vi skal prioritere.	○	○	○	○	○
7)	I afdelingen planægger vi med det rigtige antal personalemæssige ressourcer til at kunne udføre dagligdagens opgaver.	○	○	○	○	○
8)	I afdelingen er vi selvkørende og kan klare dagligdrift uden en daglig leder.	○	○	○	○	○
9)	I afdelingen oplever jeg ikke mange forstyrrelser i arbejdsdagen, som hindrer mig i at kunne udføre mit arbejde.	○	○	○	○	○
10)	I afdelingen er vi motiveret til at løse opgaver på tværs af specialer.	○	○	○	○	○

Monitoriering

		Aldrig	Sjældent	Somme tider	Ofte	Altid
11)	I afdelingen ved jeg, hvad mine kolleger laver og hvad deres kompetencer kan bruges til.	○	○	○	○	○
12)	I afdelingen kommunikerer vi tilstrækkeligt med hinanden for at sikre, at vi løser opgaverne.	○	○	○	○	○
13)	I afdelingen ved jeg, når mine kolleger er pressede og har brug for hjælp.	○	○	○	○	○
14)	I afdelingen er vi opmærksomme på løbende at forbedre arbejdsgange.	○	○	○	○	○
15)	I afdeling har vi mulighed for at få overblik over dagens arbejdsopgaver.	○	○	○	○	○
16)	I afdelingen kan jeg nemt komme i kontakt med min nærmeste leder.	○	○	○	○	○

Læring

	Aldrig	Sjældent	Somme tider	Ofte	Altid
17) I afdelingen deler vi relevant faglig viden.	○	○	○	○	○
18) I afdelingen føler jeg mig tryg ved at spørge om noget, jeg ikke kender til.	○	○	○	○	○
19) Jeg får brugbare svar på mine spørgsmål.	○	○	○	○	○
20) I afdelingen har jeg tilstrækkeligt støtte og opbakning til at udvikle mig (via nye arbejdsopgaver, træning, uddannelse, øget ansvar mv.)	○	○	○	○	○
21) I afdelingen bruger vi vores erfaringer fra gode patientforløb til læring.	○	○	○	○	○
22) I afdelingen har vi tilstrækkeligt tid at følge op på indsatser og få læring ud af det.	○	○	○	○	○

Forudsige

	Aldrig	Sjældent	Somme tider	Ofte	Altid
23) I afdelingen har vi de kompetencer, der skal til for at udføre vores arbejde.	○	○	○	○	○
24) I afdelingen er vi opmærksomme på hvor vi har udfordringer.	○	○	○	○	○
25) I afdelingen har vi fokus på at identificere fremtidige muligheder.	○	○	○	○	○
26) I afdelingen arbejder vi aktivt med at forbedre vores arbejde med henblik på fremtidige udfordringer og krav.	○	○	○	○	○
27) I afdelingen kommunikeres fremtidige planer til personalet tydeligt.	○	○	○	○	○

Kommentar

28) Har du nogen afsluttende kommentarer / uddybninger?
