# Supplementary material for: The resilient potential behaviours in an Internal Medicine Department: Application of resilience assessment grid
Source: PLoS One. 2022 Oct 17;17(10):e0276178. doi: 10.1371/journal.pone.0276178 (PMC9576065; doi:10.1371/journal.pone.0276178)
Supplement: S3 Appendix — (DOCX) [file pone.0276178.s003.docx]

**The translated English version of the RAG questionnaire**

The participants rated each question on a 5-point Likert scale (1-5):
never, rarely, sometimes, often or always

| What is your role in the Internal Medicine Department?  Manager  Nurse  Physician |
| --- |
| How many years have you worked at the Internal Medicine Department? |
| **Respond**  *R1_Flexibility:* There is time flexibility in my ambulatory program.  *R2_Teamwork:* In the department, we help each other in stressful/ under pressure situations.  *R3_Leveraging Knowledge:* In the department, we can handle/undertake each other's functions within the same professional group.  *R4_Shared priorities:* In the department, we have a common understanding of what we should prioritise.  R1_Flexibility: There is time flexibility in my ambulatory program.  R2_Teamwork: In the department, we help each other in stressful/ under pressure situations.  R3_Leveraging Knowledge: In the department, we can handle/undertake each other's functions within the same professional group.  R4_Shared priorities: In the department, we have a common understanding of what we should prioritise. R5_Ressources: In the department, we plan with the right number/amount of human resources to be able to perform everyday tasks.  R6_Self-managed: In the department, we are self-managed and can handle daily operations without a daily manager.  R7_ Interruptions: In the department, I do not experience many interruptions in the everyday work that prevent me from being able to perform my work.  R8_staff engagement: In the department, we are motivated to solve tasks across specialties. |
| **Monitor**  M1_role and responsibility: In the department, I know what my colleagues are doing and what their competencies can be used for.  M2_Communications: In the department, we communicate with each other to ensure that we solve the tasks. M3_Situation awareness: In the department, I know when my colleagues are under pressure and need help. M4_Evaluation: In the department, we are aware of continuously improving workflows.  M5_Organisational support: In the department, we have the opportunity to get an overview of the day's work tasks.  M6_Leadership: In the department, I can easily get in touch with my immediate manager. |
| **Learn**  L1_ Knowledge Dissemination: In the department, we share relevant professional knowledge.  L2_safety culture: In the department, I feel safe asking about something I do not know.  L3_relevance: I get useful answers to my questions.  L4_Development: In the department, I have sufficient support to develop or improve myself (through new work assignments, training, education, increased responsibility, etc.)  L5_Learning from what goes well: In the department, we use our experiences from good patient cases to learn.  L6_Feedback process: In the department, we have sufficient time to follow up on efforts and learn from it. |
| **Anticipate**  A1_Expertise: In the department, we have the competencies needed to carry out our work.  A2_Valnerable: In the department, we are aware of where we have challenges.  A3_Opportunistic mindset: In the department we have focus on identifying future opportunities A4_Proactiv: In the department, we work actively to improve our work with a view to future challenges and requirements.  A5_Communication: In the department, plans are clearly communicated to staff. |
| Do you have any concluding comments / elaborations? |
