# Supplementary material for: The resilient potential behaviours in an Internal Medicine Department: Application of resilience assessment grid
Source: PLoS One. 2022 Oct 17;17(10):e0276178. doi: 10.1371/journal.pone.0276178 (PMC9576065; doi:10.1371/journal.pone.0276178)
Supplement: Attachment — Submitted filename: Response to reviewers.docx [file pone.0276178.s004.docx]

Dear Editor and reviewers,

On behalf of my co-authors, many thanks for taking the time to provide constructive comments and enabling us to submit a revised manuscript. We are pleased to know that the reviewers find the study on organisation resilience using the Resilience Assessment Grid relevant and methodologically sound.

We have addressed each point raised by the reviewers. We have used tracked changes to indicate the amendments in the manuscript.

We believe these revisions have resulted in a significantly improved manuscript. We outline in detail in the document “Response to Reviewers” how we have handled each of the reviewers’ comments.

We look forward to hearing back from you.

Best regards,

Mariam Safi on behalf of all the co-authors

**Response to Journal requirements:**

1. Formatted the manuscript to meet the PLOS ONE’s style requirements (figures, tables, headings etc.). Used PACE to format the figures.
2. Funding statement and role is included in the cover letter above.
3. Data availability statement included in the cover letter above.
4. Ethic statement included in the method section.
5. Figure 1. (previously fig. 2) was referenced in the text.
6. Figure 4 (previously figure 5) was labelled as table 3 in error. This has been corrected.
7. Table 5 was referenced in the text.

**Response to reviewers:**

**Reviewer #1:** Thank you for sending me the manuscript entitled “The resilient potential behaviors in an Internal Medicine Department: Application of Resilience Assessment Grid” for review. The manuscript is well-written and well-organized. The following comments can help the authors to improve it.

| **Reviewer 1** | **Response to reviewers** |
| --- | --- |
| 1- In the abstract, the conclusion section needs to be revised. | Thank you for the comments. The conclusion in the abstract was revised. Please see lines 43-46. |
| 2- It is better to remove Figures 1 and 2. | Thank you for your feedback. I have deleted the previous figure 1. Organisation diagram of the internal medicine. However, after consideration we have decided to retain the previous figure 2 Timeline of the study (renamed figure 1.), because it provides an overview of how the RAG was applied continuously. Furthermore, it depicts the organisational changes taking place at the Internal Medicine Department. |
| 3- A sample of interview guideline and the questionnaire need to be presented as appendices. | Thank you for pointing this out. The original Danish questionnaire and translated copy of the questionnaire and the interview guide will be presented as appendices. See line 167 See line 245 Supporting information: Line 528 -531 |
| 4- Please add more information about the qualitative results’ trustworthiness. | Thank you for noticing this. We have added further details in the method section on how the qualitative findings were checked by a second author to ensure credibility. Please see lines 186-187.  Furthermore, we would like to point out that the qualitative results were indirectly further validated in Delphi rounds, when we went back to validate the RAG questions. |
| 5- In some parts of the manuscript the authors noted “health care professionals”. I think it is better to be more specific as for example, managers are not among healthcare professional. | Thank you for noticing this. We have amended the parts to make it more clear whom we are referring. Please see lines:  Line 35.  Line 282 Line 317 Line 404 Line 507 |
| 6- Please present the strengths of the study first and then talk about the research limitations. | Thank you for the comments. We made the amendments. Please see section 4.3 Strengths and limitations. |
| 7- What will happen to the suggested quality improvement initiatives? | Thank you for highlighting this. A steering committee has been created to investigate how these quality improvement can be implemented. A new workshop in fall 2022 will work further with the initiatives.  We have elaborated this further in section 6.2 lines 479-482. |

**Reviewer #2**: Dear authors,
Congratulations for the submission of paper entitled 'The resilient potential behaviours in an Internal Medicine Department: Application of Resilience Assessment Grid' to PLOS ONE. The organization resilience and its attempts to stay adaptive with challenging conditions while still maintaining/increasing the quality are critical issues nowadays. I appreciate the robust and systematic research methods including the questionnaire development. However, with the current form, I cannot recommend publication in the journal. I believe the authors should revise the paper substantially. There are some suggestions listed below for the authors to consider during revision:

| **Reviewer 2** | **Response to reviewer** |
| --- | --- |
| 1. Introduction The elaboration on RAG and its importance within organization is quite good and easy to understand regardless the familiarity towards the topic. It is not clear to me, however, the gap analysis of this study. Why this study should be conducted? Why concerns in the internal medicine department in one teaching hospital should become concerns of the readers coming from different settings? Therefore, I recommend a stronger and clearer gap analysis elaborating what's not yet elaborated in the literature regarding this topic. Also, what are the research questions of this study? Despite the elaboration of the aim, clearer research questions are warranted to offer clear justification of the study. Further elaboration on the response or expected results of this study is also needed to give further insights on what the study would add in the literature. | Thank you for the comments. We have revised the introduction and addressed: The gap in the literature, Lines 104-106.  Research question, lines107-108 The relevance of the study for other settings, Line 114-117.  Furthermore, we have elaborated the RAG method a little further which explains our decisions on how we developed and applied the RAG. Lines 94-98. |
| **2. Methods** The methods are described systematically. The setting which was explained in the introduction should be considered to be moved as the first section in the methods. The steps of instrument development are mostly well detailed. Yet, given issues in the introduction, the current stages are confusing. In addition, decisions on the method such as the exclusion of medical/nursing students, should be justified and explained. Each stage of study, including the delphi, the pilot of the instrument and so on should be better elaborated in terms of the data collection, sample, and the data analysis. I would expect better description on the thematic analysis being completed for the interview. I also wonder why there is no exploratory factor analysis or confirmatory factor analysis to support the construct of the instrument. Nor there was attempt to elaborate the reliability of the questionnaire - overall score, and subscale scores. I understand the value of reporting the mean score of each item as part of identifying the rooms for improvement in the system, however it is not clear to me why the subscale scores were not reported. Finally, at the presentation stage, I am quite confused with how was it done and how did this process add further data in this study? If so, how was it analyzed? | Thank you for the comments. Below we have addressed the comments regarding the method section.  **Setting:** We have moved the setting in the method section. Please see section 2.1 setting.  **Exclusion of medical/nursing students:** Please see lines 253-256 for the explanation.  **Thematic analysis** The RAG is not a typical survey, whereby the questions are developed and validated so that it can then be applied across multiple settings. Rather, it is a pragmatic tool used in industry to enable context specific understanding of the factors contributing to organisational resilience. Therefore the survey development approach is a little different from the usual qualitative study. A large-scale qualitative research is not conducted when developing the RAG questionnaire. Primarily, it is important that the questions fit the context and that they are developed in collaboration with the managers and staff. Previous studies^[1]^ ^[2]^ ^[3]^ on RAG have used an expert panel or focus group to adapt the RAG framework to the specific setting.  However, we wanted to explore the potential of developing the RAG from a research perspective, so therefore used a more robust approach. We have amended the manuscript to clarify this: Please see the track changes in section 2.2, where we have provided further details on data collection, thematic analysis and pilot testing. Lines 171 – 217.  **Pilot testing:** We have elaborated the pilot testing a little more. Please see lines 221-224  **Regarding the exploratory factor analysis**, we explored analysis options with our statistician. Because the survey is context specific and not generalisable to other organisations, and also because the survey should be able to be developed by industry practitioners who do not possess high level analysis skills, confirmatory factor analysis was considered to be inappropriate. In consultation with the statistician and a researcher in scale development, we instead computed a Content Validity Index (CVI) and Scale Content Validity (S-CVI) using the framework provided by Polit et al. Polit et al. recommend CVI as an appropriate indicator for content validity for health services research, based on a comparative evaluation of the CVI and alternative indexes.  Please see the method section lines 166-215.  The reliability of the questionnaire was ensured by the Delphi rounds and the qualitative pilot testing. The RAG questions were reviewed by the respondents and by an external resilience expert (Hollnagel, the developer of RAG). Hollnagel critically reviewed its concordance with the underlying RAG construct. Lines 215-217. We ensured that the questions had high content validity.  **Radar charts:** please see line 263 -264 on how the radar chart were illustrated.  **Subscale score for the RAG:** We have reported this in table 2. Please see the highlighted text. We agree with you that in the presentation of the results, we did not emphasis the subclass score because it does not tell much about how the system works. |
| 3. Results The results are quite well written. Yet the clarity can be further enhanced by addressing the issues in the Methods section. All collected data be it qualitative and quantitative should be presented accurately and adequately. | Thank you for your comments. We have addressed the issues in the Method section. This has further enhanced the clarity of the results. |
| 4. Discussion and conclusion The discussion is rather long. The authors may want to consider shortening the discussion section and focus more on comparing the results with the available and relevant literature as well as analyze the implications. | Thank you for the comment. After careful consideration, we have decided that we would like to retain the discussion. It is important that the major findings are discussed. Furthermore, it is highly relevant that the RAG as a method is discussed. This will enlighten the readers on how to develop and use the RAG in their setting.  Comparison of the results: see lines 419-438. Lines 448 -458.  Implication for research: lines 459 -471 |

1. Patriarca R, Di Gravio G, Costantino F, Falegnami A, Bilotta F. An analytic framework to assess organizational resilience. Safety and health at work. 2018;9(3):265-76
2. Chuang S, Ou J-C, Ma H-P. Measurement of resilience potentials in emergency departments: Applications of a tailored resilience assessment grid. Safety Science. 2020;121:385-93.
3. Hunte G, Marsden J. Engineering resilience in an urban emergency department. Delivering Resilient Health Care: Routledge; 2018. p. 131-49.
